# Immune dysregulation in mania: A proof‐of‐concept platelet proteomics study

**DOI:** 10.1111/pcn.70055

**Published:** 2026-04-04

**Authors:** Paola Magioncalda, Matteo Martino, Nhi Thao Ngoc Le, Wen‐Yin Chen, Ming‐Chyi Huang, David Blum, Thierry Burnouf

**Affiliations:** ^1^ International Master/Ph.D. Program in Medicine, College of Medicine Taipei Medical University Taipei Taiwan; ^2^ Graduate Institute of Mind Brain and Consciousness Taipei Medical University Taipei Taiwan; ^3^ International PhD Program in Biomedical Engineering, College of Biomedical Engineering Taipei Medical University Taipei Taiwan; ^4^ Taipei City Psychiatric Center (TCPC) Taipei City Hospital Songde Branch Taipei Taiwan; ^5^ School of Medicine, College of Medicine Fu Jen Catholic University New Taipei City Taiwan; ^6^ University of Lille, Inserm, CHU Lille, UMR‐S1172 Lille Neuroscience & Cognition (LilNCog) Lille France; ^7^ Graduate Institute of Biomedical Materials and Tissue Engineering, College of Biomedical Engineering Taipei Medical University Taipei Taiwan

**Keywords:** bipolar disorder, mania, MHC, platelets, proteomics

## Abstract

**Aims:**

Bipolar disorder (BD) is a major psychiatric condition with a multifaceted and largely unknown pathophysiology. Mania, the defining feature of BD, remains underinvestigated. Proteomics offers a powerful, data‐driven, unbiased approach to uncover biological alterations. Blood platelets provide a stable and comprehensive reflection of the body's internal milieu. This proof‐of‐concept study explores platelet proteomic alterations in mania.

**Methods:**

High‐resolution proteomic profiling was performed on platelet samples from hospitalized patients with severe mania (*n* = 11) and 1:1 age‐ and sex‐matched healthy controls (*n* = 11) using liquid chromatography–tandem mass spectrometry. Platelet proteins were quantified, differentially expressed proteins identified, and functional enrichment analyses conducted to characterize associated biological pathways. Key proteomic predictions were validated by enzyme‐linked immunosorbent assay (ELISA) in platelet and plasma samples.

**Results:**

Proteomic analysis revealed that mania is associated with proteins primarily enriched in immune activation while lacking those enriched in cell homeostasis. Among shared proteins, mania exhibited a prominent cluster of downregulated proteins, primarily converging on immune‐related pathways. The most robust alteration involved deficits in MHC Class I‐mediated antigen processing. Key immune regulatory hubs – transforming growth factor (TGF)‐β and interleukin‐4 (IL‐4) – were identified, with the association of increased TGF‐β levels with mania validated by ELISA.

**Conclusions:**

This study highlights platelet proteomics as a valuable tool for investigating biological alterations in psychiatric disorders. Our findings indicate deficits in MHC Class I‐related pathways and immune alterations consistent with chronic low‐grade inflammation, suggesting potential roles for latent viral and autoimmune‐related mechanisms in BD. These results support and refine the concept of immune dysregulation in mania and BD.

Bipolar disorder (BD) is a major psychiatric condition clinically characterized by recurrent episodes of mania and depression, alternating with asymptomatic periods of euthymia.^1^ In particular, mania is an excited state featuring abnormally elevated or irritable mood, increased energy and activity levels, and impulsive behavior.^1^ Conversely, depression exhibits depressed mood or anhedonia, accompanied by a heterogeneous constellation of cognitive, psychomotor, and vegetative symptoms, such as depressive rumination, impaired concentration or indecisiveness, psychomotor retardation or agitation, insomnia or hypersomnia, and weight loss or gain.^1^ Despite extensive research, the pathophysiology of BD remains largely unknown, with often heterogeneous and inconsistent findings.

This may reflect the multifaceted nature of BD, including limitations inherent in current clinical conceptualizations and research approaches. Most studies examine BD as a unitary condition, irrespective of illness phase, primarily focusing on the euthymic state or, more often, the depressive phase, which has dominated the field in recent decades due to its higher prevalence, longer duration, greater subjective burden, and increased resistance to treatment.^2^, ^3^ However, the multiform clinical presentation of BD – especially the depressive phase, which encompasses a broad and heterogeneous set of symptoms – may contribute to the variability and the inconsistency observed regarding biological findings. Conversely, mania, which is the defining criterion for BD in current psychiatric classifications, features a more uniform and specific symptom pattern.^1^ Importantly, classical psychopathological models suggest an intrinsic, structural link between mania and depression, conceptualizing them as components of a manic‐depressive cycle, with mania serving as the core pathophysiological process and depression as a downstream consequence (‘*mania is the fire, depression is its ashes*’).^2^, ^4^ Therefore, investigating mania may yield more consistent results and help isolate key biological factors that could not only deepen our understanding of the biological basis of mania itself but also prove critical for elucidating the overall pathophysiology of BD, including depressive states.^2^ However, despite its clinical relevance and distinct features, research specifically focused on mania remains limited.

Another key issue is the inherent complexity of BD pathophysiology. The biological underpinnings of BD involve alterations in brain structure and function, including prominent disruptions in white matter microstructure, changes in neurotransmitter signaling, and abnormalities in the functional architecture of brain activity.^5^, ^6^, ^7^, ^8^, ^9^ Notably, various systemic abnormalities have also been reported, with chronic low‐grade inflammation being among the most consistently observed.^10^, ^11^ For instance, levels or activity of pro‐inflammatory cytokines and other soluble factors – such as interleukin (IL)‐6, tumor necrosis factor (TNF)‐α, and C‐reactive protein (CRP) – have consistently been found to be elevated in BD, particularly during the manic phase.^10^, ^11^ The origins of this systemic inflammation remain elusive, and the interplay among the various biological alterations appears fragmented. As a result, it remains unclear whether BD should be conceptualized as a primary brain disorder, a systemic condition affecting the brain, or a combination of both. A more systematic investigation of the body's internal milieu may help identify peripheral signatures associated with BD and contribute to a more integrated understanding of its pathophysiology.

In this context, high‐resolution proteomics has recently emerged as a powerful tool for investigating biological alterations through a data‐driven, unbiased, and hypothesis‐free approach.^12^ In particular, untargeted quantitative proteomics enables the identification, characterization, and quantification of the relative abundance of the complete set of proteins expressed by a cell, tissue, or organism at a specific time, referred to as the proteome.^12^ As the dynamic expression of proteins reflects ongoing biological processes and responds to developmental, environmental, and pathological factors, the proteome represents a potential signature of health and disease states.^12^ While more invasive approaches – such as analyses of postmortem brain tissue and cerebrospinal fluid – have been employed to investigate molecular alterations in BD, blood remains the most commonly studied and available material in proteomic research due to its accessibility and ability to reflect systemic physiological states. In particular, recent investigations of the plasma and serum proteome in BD have revealed heterogeneous protein alterations, enriched in pathways related to complement and coagulation cascades, lipid and cholesterol metabolism, and focal adhesion.^13^ However, whether in plasma, serum, or even cerebrospinal fluid, this type of analysis captures only a snapshot of physiological and pathological states at the time of sample collection, and the pathological significance of the observed changes remains unclear.^14^

Interestingly, an alternative source of information is offered by a frequently overlooked blood component: platelets. These small, anucleate cytoplasmic fragments derived from megakaryocytes, primarily known for their role in hemostasis, also offer a valuable tool for investigating the body's internal milieu.^14^ Through their open canalicular system, which mediates active uptake and release of signaling molecules, as well as various membrane receptors, platelets act as dynamic reservoirs of bioactive molecules and neuroactive compounds.^14^ Over their 7–10‐day lifespan, they can capture and retain systemic biological signals, making them stable peripheral biomarkers.^14^ Consequently, platelet proteomics can capture integrated biological responses over the platelet's lifespan, offering a retrospective and more stable view of physiological and pathological states.^14^ Beyond hemostasis, platelets actively participate in inflammation, immune modulation, and signaling across the blood–brain barrier,^14^, ^15^, ^16^, ^17^ positioning them as promising peripheral markers for psychiatric conditions with a significant immune‐inflammatory component, such as BD. Platelet proteomics, therefore, may represent a privileged tool to investigate the biology of BD, potentially revealing systemic, pathologically meaningful alterations that are otherwise inaccessible through more conventional approaches. Despite this potential, no studies to date have applied this strategy to BD, or even, more broadly, to any other psychiatric disorder.

The present proof‐of‐concept study aims at addressing this gap by investigating the platelet proteome in BD. Specifically, it explores the body's internal milieu in a robust, in‐depth, data‐driven, and hypothesis‐free manner using high‐resolution platelet proteomics in mania, the core and more homogeneous phase of BD.

## Methods

### Subjects and clinical assessment

Patients were recruited from the in‐patient department of a tertiary‐level psychiatric hospital (Taipei City Hospital SongDe Branch, Taipei, Taiwan), while healthy controls (HC) were from the metropolitan area of Taipei, Taiwan. The study was approved by the Research Ethics Committee of Taipei City Hospital and the Institutional Review Board of Taipei Medical University. Written informed consent was obtained from all participants. The study was conducted on 11 patients with BD type I during a manic episode, along with 11 HC individually matched for age and sex (1:1 matching).

Each participant was evaluated by a board‐certified expert psychiatrist using standardized clinical instruments to assess clinical and diagnostic features, illness course, and pharmacotherapy: Mini International Neuropsychiatric Interview (MINI),^18^ Young mania rating scale (YMRS),^19^ and Hamilton depression rating scale (HAM‐D) with 17 items.^20^ A comprehensive psychiatric evaluation was conducted, encompassing general, physiological, pathological, and psychopathological history.

Inclusion criteria were: a diagnosis of BD type I according to DSM‐5 criteria^1^; current manic episode (with YMRS score >13); age between 21 and 60 years; and the ability to provide written informed consent. Exclusion criteria were: a diagnosis of other major neuropsychiatric disorders (e.g., schizophrenia, intellectual disability, or neurocognitive disorders); current alcohol or substance abuse (within the past 3 months) or a history of addiction; severe or decompensated medical and/or neurological conditions; clinically manifest infections, autoimmune disorders or immune‐inflammatory disorders; platelet or coagulation disorders, daily use of low‐dose aspirin or treatment with heparin; and pregnancy. HC did not meet DSM criteria for any psychiatric disorder, either at the time of participation or in the past. They had YMRS and HAM‐D scores equal to 0 and met the same exclusion criteria applied to patients.

### Platelet and plasma sample preparation

#### Blood sample collection

A total of 10 mL of blood was collected into tubes containing a citrate anticoagulant solution. The samples were centrifuged within 2 h of collection at 300 x g for 5 min at 20–25°C to sediment red blood cells. The platelet‐rich plasma (PRP) supernatant was carefully recovered and further centrifuged at 3,000 x g for 15 min at 20–25°C. The supernatant (platelet‐poor plasma, PPP) was collected and stored at −80°C. The surface of the platelet pellet was gently washed with phosphate‐buffered saline (PBS) to remove any remaining PPP and subsequently stored at −80°C.

#### Platelet protein extraction

Frozen platelet pellets were resuspended in half of their original PRP volume using PBS. The samples were subjected to two additional freeze–thaw cycles at −80/37°C to lyse the platelet membranes and release the platelet contents (platelet pellet lysate, PPL).^21^ Afterward, the samples were centrifuged at 6,000 x g for 20 min at 22°C to pellet the cell debris. The supernatant containing the PPL was recovered for subsequent analyses.

#### Bicinchoninic acid assay

Total protein content was measured using the bicinchoninic acid (BCA) Protein Assay Kit (Thermo Scientific), with a bovine serum albumin (BSA) calibration curve. A 25‐μL sample was mixed with 200 μL of BCA reagent and incubated at 37°C for 30 min. Absorbance was then recorded at 562 nm using a microplate spectrophotometer (Agilent BioTek Epoch 2, Santa Clara, CA, USA).

### Proteomics analysis

#### Sample preparation for label‐free proteomics

PPL samples were precipitated with pre‐cooled acetone at a 1:4 (v/v) ratio and incubated overnight at −20°C. After centrifugation (15,000 x g, 10 min, 4°C), the protein pellet was washed twice with cold acetone (1:4), followed by centrifugation (13,000 x g, 10 min, 4°C), air‐drying, and resuspension in 6M urea. Protein content was determined using the BCA assay, and 20 μg of protein was reduced with 5 mM dithiothreitol (29°C, 45 min, in the dark), alkylated with 10 mM iodoacetamide (29°C, 45 min, in the dark), and digested with trypsin (1:50 ratio, 29°C, 16 h). The digestion was stopped with 0.5% trifluoroacetic acid, and peptides were purified using C18 Zip‐Tip.

#### Mass spectrometry data acquisition

Quantitative label‐free proteomics was performed using liquid chromatography–tandem mass spectrometry (LC–MS/MS) on an Orbitrap Fusion Lumos Tribrid Mass Spectrometer (Thermo Fisher Scientific, San Jose, CA, USA). Peptides were separated on a Dionex Ultimate 3000 nanoLC system with 2‐μm particles and 100 Å pores. Data‐dependent acquisition mode was employed, with a full‐MS resolution of 120,000 at *m/z* 200. Raw data were analyzed using Proteome Discoverer 2.2 and searched against the UniProt Swiss‐Prot human database (August 2017) via Mascot, with a false discovery rate (FDR) threshold of <1%. The search parameters included trypsin digestion (≤2 missed cleavages), MS tolerance (10 ppm), MS/MS tolerance (0.02 Da), and modifications for oxidation (M), deamidation (NQ), carbamidomethylation (C), phosphorylation (STY), acetylation (K), and methylation (K). Only high‐confidence proteins with a Mascot score ≥30 were considered.

#### Bioinformatics analysis

Protein abundances were normalized by reporter‐to‐total intensity. To visualize patterns and correlations in the proteomics data, principal component analysis (PCA) and partial least squares‐discriminant analysis (PLS‐DA) were performed using Scikit‐learn.

Proteins were subsequently quantified only if they were detected in at least 8 out of 11 samples (≥75%) within a group. This threshold was chosen to ensure reliability, minimize the impact of missing values, and retain consistently observed proteins.

Gene ontology (GO) annotation was performed using DAVID (https://david.ncifcrf.gov/) on the proteins quantified in all samples, as well as those uniquely quantified in either manic patients or HC. This provided insights into their molecular functions, involvement in biological processes, and cellular localization within and across groups.

Among the proteins quantified in both groups, differentially expressed proteins (DEPs) were identified by first performing an *F*‐test to assess the equality of variances. If the *F*‐test *P*‐value exceeded 0.05, indicating equal variances, a pooled *t*‐test was applied; otherwise, Welch's *t*‐test was used to account for unequal variances. Proteins with *P* <0.05 and fold‐change ≥1.2 were considered significantly differentially expressed. A Venn diagram was generated using matplotlib‐venn to illustrate the overlap and uniqueness of proteins between groups.

To explore the underlying structure of DEPs, unsupervised hierarchical clustering was performed on z‐scored protein abundance using the ComplexHeatmap R package, with clustering based on Euclidean distance and complete linkage.

Molecular function‐based categorization of the DEPs was also conducted to identify overrepresented functional categories among the significantly altered proteins.

Ingenuity pathway analysis (IPA, QIAGEN, USA) was used for functional annotation, canonical pathway analysis, and core network exploration of DEPs. The analysis followed a data‐driven, unbiased approach, without pre‐filtering for specific pathways or functions.

Finally, protein–protein interaction (PPI) networks of DEPs were analyzed using IPA Core Analyze, identifying potential functional hubs and interaction patterns.

### Enzyme‐linked immunosorbent assay analysis

As post hoc analyses, proteins identified as central regulatory hubs in the PPI analysis of the proteomics data were subsequently measured in platelets and plasma to validate their involvement in the proteomic alterations associated with mania. Plasma and platelet (PPL) levels of the selected proteins were quantified using enzyme‐linked immunosorbent assay (ELISA) kits from R&D Systems (Cat# DY240‐05, Minneapolis, USA) and Abcam (Cat# ab46058, Cambridge, UK), respectively, following the manufacturers' instructions. Optical density was recorded within 30 min using a microplate reader. Protein levels were normalized to total protein content to account for differences in sample loading. All assays were performed by a technician blinded to sample identity and clinical information.

Differences in platelet and plasma levels of the tested proteins between manic patients and HC were assessed using the Wilcoxon signed‐rank test, given the paired design and small sample size. Statistical significance was set at *P* <0.05. Analyses were performed using GraphPad Prism 9.0 (La Jolla, CA, USA).

Finally, clinical relationships between platelet and plasma levels of the tested proteins and manic symptomatology were explored. Given the small sample size and the ordinal nature of the YMRS, associations between protein levels and YMRS scores were assessed using Kendall's tau‐b correlations. Scatterplots were visually inspected for potential influential observations.

## Results

### Clinical description of the sample

The sample included 11 patients diagnosed with BD type I during a severe manic episode (mean age = 41.4 ± 13.7 years; 7 females) and 11 HC matched 1:1 for age and sex (mean age = 40.9 ± 13.1 years; 7 females). All manic patients were hospitalized in a tertiary‐level psychiatric facility at the time of study enrollment. The mean YMRS score was 23.64 (±4.20), with only one patient scoring below 20 and 5 patients scoring above 26. Nine patients exhibited psychotic symptoms, predominantly grandiose delusions. All patients were under pharmacological treatment with mood stabilizers, antipsychotics, and benzodiazepines. None of the patients had any relevant psychiatric comorbidities. These characteristics indicate that the sample represents a homogeneous and well‐defined group of individuals with pure mania. A detailed description of the sample can be found in Table 1.

**Table 1:** Subject demographic and clinical information

	M	HC
Sample size, *n*	11	11
Age, *mean (SD)*	41.4 (13.7)	40.9 (13)
Female, *n (%)*	7 (63.6)	7 (63.6)
YMRS, *mean (SD)*	23.6 (4.2)	
HAM‐D, mean (SD)	3.9 (2.5)	
Psychotic features (grandiose delusions), *n (%)*	9 (81.8)	
Age of onset, *mean (SD)*	31.2 (12.9)	
Duration of illness (years), *mean (SD)*	10.3 (12.1)	
Number of previous total episodes, *mean (SD)*	6.3 (5.7)	
Number of previous manic episodes, *mean (SD)*	5.7 (5.3)	
Number of previous depressive episodes, *mean (SD)*	0.5 (1)	
Number of hospitalizations, *mean (SD)*	5 (4.9)	
Mood stabilizers, *n (%)*	11 (100)	
Antipsychotics, *n (%)*	10 (90.9)	
Antidepressants, *n (%)*	0	
Benzodiazepines, *n (%)*	10 (90.9)	

M, mania; HC, healthy controls; YMRS, Young mania rating scale; HAM‐D, Hamilton depression scale; SD, standard deviation.

### Platelet proteomic profile

**Fig. 1:**
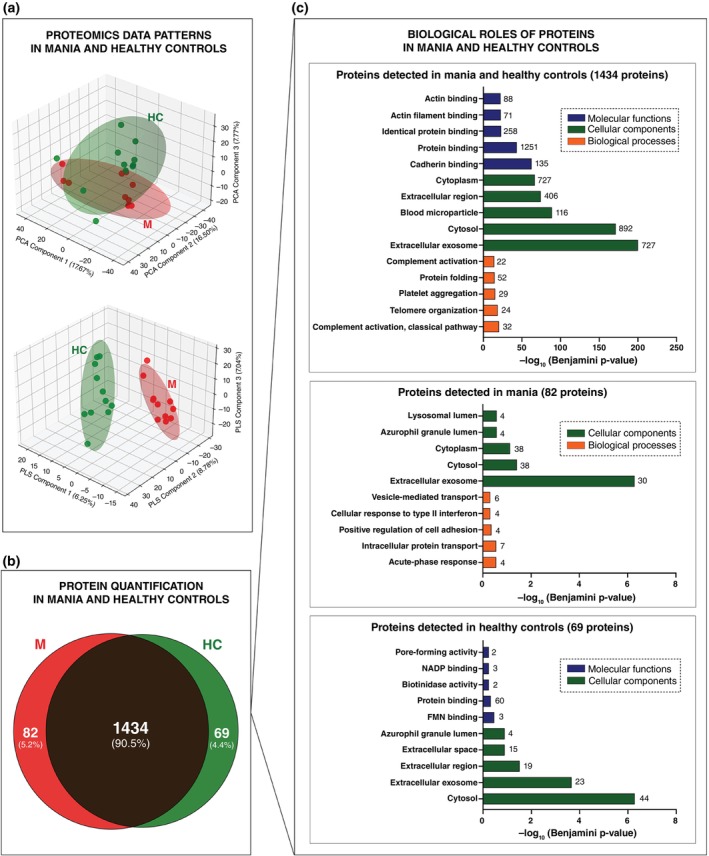
Platelet proteomic profile in mania and healthy controls. (a) PCA plot showing proteomics variation between groups; PLS‐DA plot highlighting distinct clustering of manic patients and healthy controls. (b) Venn diagram illustrating the shared and unique proteins in manic patients and healthy controls. (c) GO‐based functional enrichment analysis of proteins quantified in both groups, as well as those unique to manic patients and healthy controls; proteins are categorized into molecular functions, cellular components, and biological processes, with numbers near each bar indicating the count of proteins associated with each GO term. M, mania; HC, healthy controls; PPL, platelet pellet lysate; PCA, principal component analysis; PLS‐DA, partial least squares‐discriminant analysis; GO, gene ontology.

PCA indicated partial overlap between manic patients and HC, suggesting shared molecular features alongside group‐specific differences. PLS‐DA further confirmed that the groups are significantly separable when group labels are considered, highlighting distinct platelet proteomic signatures. See Figure 1a.

A total of 1,503 proteins in manic patients and 1,516 in HC met the ≥75% predefined detection threshold and were included in quantitative analyses. Of these, 1,434 proteins (90.5%) were quantified in both groups, indicating substantial molecular overlap. Interestingly, 82 proteins (5.2%) were quantified in manic patients only and 69 proteins (4.4%) were quantified in HC only. See Figure 1b.

GO analysis of the quantified proteins classified them into molecular functions, cellular components, and biological processes. Proteins shared between manic patients and HC were enriched in molecular functions related to cadherin binding (implicated in cell–cell interactions), protein binding, and actin binding. The main associated cellular components included extracellular exosomes, cytosol, blood microparticles, extracellular region, and cytoplasm. Enriched biological processes included complement activation, telomere organization, platelet activation (suggesting involvement in hemostatic regulation), and protein folding (indicating a role in proteostasis). Proteins uniquely quantified in manic patients were enriched in additional cellular components, such as the lysosomal lumen, and biological processes including intracellular protein and vesicle‐mediated transport, positive regulation of cell adhesion, cellular response to interferon, and acute‐phase response (indicating cellular stress response and immune activation). In contrast, proteins uniquely quantified in HC were enriched in additional cellular components, such as the extracellular space, and molecular functions including nicotinamide adenine dinucleotide phosphate (NADP) and flavin mononucleotide (FMN) binding, pore‐forming activity, and biotinidase activity (involved in cellular metabolism and membrane integrity). See Figure 1c.

### Functional and pathway profile of differentially expressed platelet proteins in mania

**Fig. 2:**
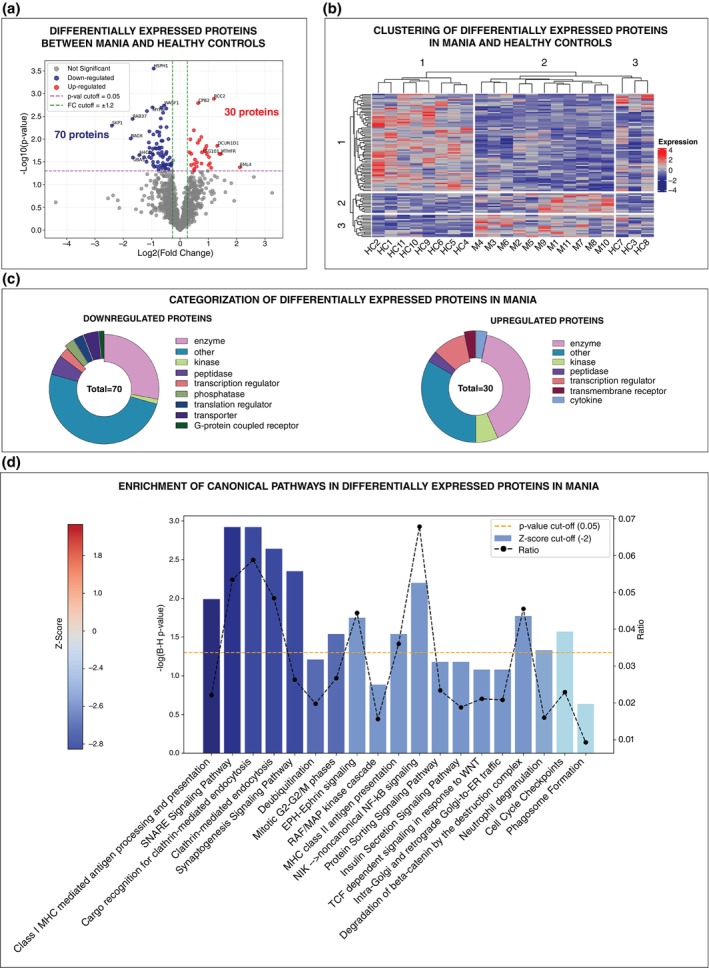
Functional and pathway profile of differentially expressed platelet proteins in mania and healthy controls. (a) Volcano plot identifying DEPs, highlighting significantly downregulated and upregulated proteins. (b) Heatmap showing hierarchical clustering of DEPs based on expression levels. (c) Molecular classification of downregulated and upregulated DEPs by functional type. (d) Canonical pathway analysis illustrating enriched biological pathways associated with DEPs. M, mania; HC, healthy controls; DEPs, differentially expressed proteins.

Comparison of the 1,434 proteins shared between groups revealed DEPs between manic patients and HC, with 70 proteins downregulated and 30 upregulated in mania, as illustrated by the volcano plot in Figure 2a.

Unsupervised clustering of DEPs revealed a distinct proteomic profile in manic patients, clearly separating them from HC. A prominent cluster of downregulated proteins emerged, suggesting reduced expression of specific proteins in mania, as visualized in the heatmap in Figure 2b.

Molecular function‐based categorization of both downregulated and upregulated proteins in mania indicated that enzymes constituted the predominant class among DEPs, followed by less represented categories such as peptidases, transcription regulators, and kinases. Transporters, phosphatases, translation regulators, and G‐protein‐coupled receptors were most likely downregulated, whereas cytokines and transmembrane receptors were predominantly upregulated. See Figure 2c.

IPA of DEPs revealed significant enrichment of canonical pathways downregulated in mania. These included immune‐related pathways (MHC Class I and II‐mediated antigen processing and NF‐κB signaling), intracellular trafficking (SNARE signaling and clathrin‐mediated endocytosis), neurodevelopmental processes (synaptogenesis, EPH–Ephrin signaling, and mitotic G2/M phase transition), and protein metabolism (e.g., β‐catenin degradation). Notably, MHC Class I antigen presentation showed the most strongly negative activation z‐score, indicating marked inhibition of this pathway and a potential deficit in antigen processing and presentation in mania. See Figure 2d.

### Network profile of differentially expressed platelet proteins in mania

**Fig. 3:**
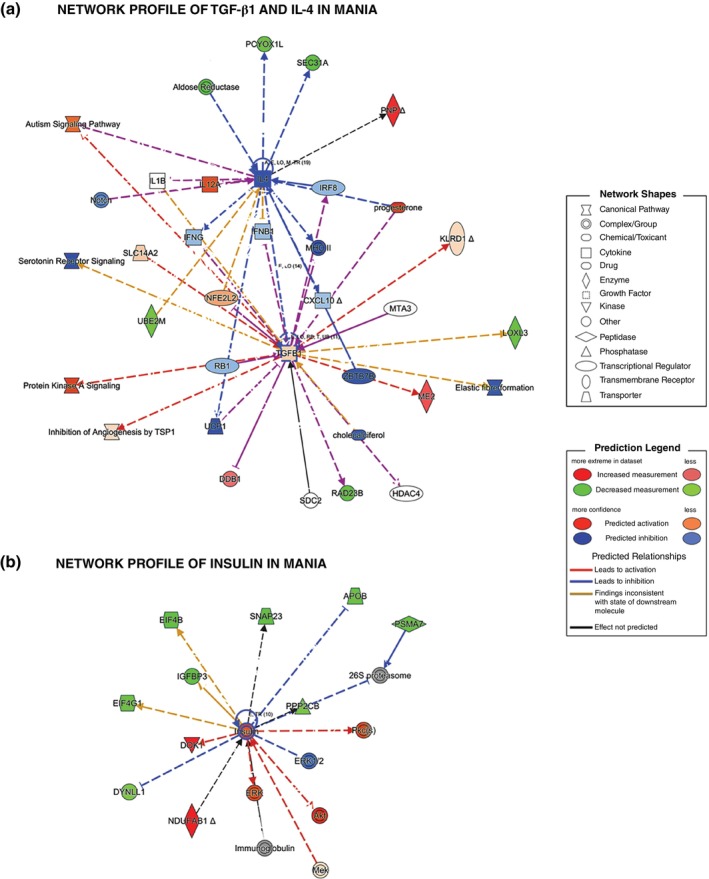
Network profile of differentially expressed platelet proteins in mania. PPI network analysis identifying key regulatory hubs that connect altered pathways of DEPs: (a) cytokine‐centered network; (b) insulin‐centered network. PPI, protein‐protein interaction; DEPs, differentially expressed proteins; TGF‐β1, transforming growth factor beta 1; IL‐4, interleukin 4.

PPI network analysis revealed how DEPs interact with one another, highlighting key hub proteins (i.e., highly connected nodes) that integrate multiple signaling pathways and play central roles in cellular signaling and regulation. To ensure an unbiased representation, pathways were selected based on predefined statistical criteria rather than subjective interpretation. Using this approach, transforming growth factor (TGF)‐β1, IL‐4, and insulin emerged as central regulatory hubs linking the altered pathways of DEPs. In particular, TGF‐β1 was predicted to be activated, whereas IL‐4 was predicted to be inhibited. These cytokine changes were associated with predicted inhibition of interferon (IFN)‐γ and IFN‐β, activation of IL‐12, and modulation of IL‐1 signaling. Notably, cytokine changes – particularly TGF‐β1 upregulation – were associated with predicted inhibition of serotonin receptor signaling, elastic fiber formation, and angiogenesis, alongside activation of protein kinase A and autism‐related signaling pathways. Finally, insulin was predicted to be upregulated. Please note that the network was faded for distant nodes to emphasize those directly connected to the main regulatory hubs. See Figure 3.

### Validation of network‐predicted cytokine alterations in mania

**Fig. 4:**
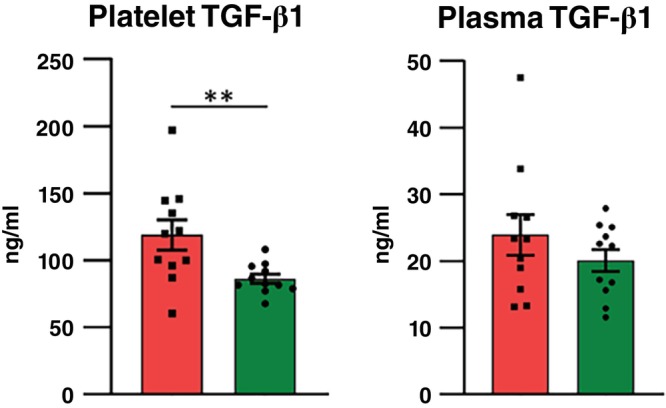
Validation of network‐predicted cytokine alterations in mania. ELISA‐based quantification and comparison of TGF‐β1 levels between manic patients and healthy controls using one‐tailed Wilcoxon signed‐rank test. Platelet levels of TGF‐β1 are significantly elevated in manic patients compared to healthy controls (*W* = −58; *P* = 0.003). Plasma levels show no significant differences between groups (*W* = −20; *P* = 0.2). Data are presented as mean ± standard error. **P < 0.01. M, mania; HC, healthy controls; TGF‐β1, transforming growth factor beta 1.

As revealed by the network analysis, TGF‐β1, IL‐4, and insulin emerged as key hub proteins integrating the altered pathways among DEPs in mania and were predicted to be dysregulated. To validate the immune‐related predictions, ELISA analyses were conducted for TGF‐β1 and IL‐4 in both platelets and plasma. Consistent with the PPI‐based predictions, platelet levels of TGF‐β1 were significantly elevated in manic patients compared to HC; conversely, no significant group differences were observed in plasma levels. In contrast, IL‐4 levels in both platelet and plasma samples were below the detection threshold in the majority of subjects, precluding statistical comparison between groups. See Figure 4.

Finally, exploratory analyses examining the relationship of platelet and plasma TGF‐β1 levels with manic symptomatology revealed a significant association between plasma TGF‐β1 and YMRS Item 1 (elevated mood) (*τ* = 0.63, *P* = 0.016, uncorrected). No other significant correlations were observed. See Table S1.

## Discussion

### Main findings

This work represents a proof‐of‐concept for a systematic, data‐driven, in‐depth characterization of the platelet proteome – a fundamental proxy for the body's internal milieu – in mania, the core of BD.

First, the analysis revealed that mania was associated with a distinct protein cluster primarily enriched in immune activation and cellular stress response (such as acute‐phase reaction, interferon responses, and cell adhesion), while lacking a protein cluster primarily enriched in cellular metabolism and membrane integrity (such as NADP and FMN binding, pore‐forming activity, and biotinidase activity). Second, among the shared proteins, mania showed a prominent set of downregulated proteins – primarily enzymes, proteases, and signaling and regulatory proteins – converging predominantly on immune‐related pathways. Notably, the most significant deficit was observed in MHC Class I‐mediated antigen processing. Additional changes affected pathways related to cell maturation and homeostasis, particularly those involving neural processes. Third, key regulatory hubs in these dysregulated pathways in mania were the cytokines TGF‐β and IL‐4, along with insulin. Finally, validating the proteomic findings, TGF‐β was associated with mania, with platelet levels elevated in manic patients and plasma levels correlated with mood elevation.

These findings demonstrate the feasibility and relevance of using platelets – immune‐competent, sensor‐rich blood components – as a minimally invasive window into systemic alterations linked to psychiatric conditions. Taken together, platelet proteome alterations in mania converge on immune dysregulation.

### Immune dysregulation in mania

This study, to the best of our knowledge, represents the first platelet proteome analysis in psychiatric disorders. In this context, our findings provide the first evidence of altered protein expression in the MHC Class I pathway in mania and BD. Prior robust work has demonstrated a polygenic architecture in major psychiatric disorders, with the MHC notably emerging as the most strongly associated locus in schizophrenia and showing significant polygenic overlap with BD.^22^ Thus, our preliminary findings on the functional downregulation of proteins involved in MHC antigen processing in mania complement this robust genetic evidence and suggest that platelet‐based proteomics may serve as a viable surrogate to investigate immune dysfunction in psychiatric disorders, with translational potential for biomarker development. Together, these data point to a potential key role for MHC‐related immune dysfunction in the pathophysiology of major psychiatric disorders, including BD.

Physiologically, the main function of MHC molecules is to present antigenic fragments to the immune system.^23^ MHC Class I molecules, expressed by all nucleated cells, present endogenous (intracellular) peptides to CD8+ cytotoxic T cells.^23^ These antigens are processed by cytosolic and nuclear proteases, and the resulting peptides are transported into the endoplasmic reticulum by transporter associated with antigen processing to bind MHC I molecules at cell surface.^23^ MHC I constantly displays self‐peptides, enabling immune tolerance, while non‐self‐peptides – derived from viruses, intracellular bacteria, or tumor cells – activate CD8+ T cells to eliminate infected or abnormal cells.^23^ This pathway is central to immune surveillance against intracellular pathogens, like viruses, and tumors.^23^ In contrast, MHC Class II molecules, primarily expressed by antigen‐presenting cells (e.g., dendritic cells, macrophages, B cells), present exogenous peptides to CD4+ helper T cells, playing a key role in immunity against extracellular organisms such as bacteria and fungi.^23^ Notably, MHC deficits have been robustly linked to increased susceptibility to viral infections, autoimmune disorders, and chronic inflammation.^23^, ^24^, ^25^ Accordingly, the deficits observed here in MHC‐related pathways – particularly within the MHC Class I pathway – in mania raise the hypothesis that this immune alteration might increase susceptibility to intracellular pathogens (such as viruses) and alter self/non‐self discrimination (promoting autoimmune mechanisms), as potential contributors to the pathophysiology of BD.

In line with these considerations, proteins uniquely quantified in mania were primarily enriched in immune activation and cellular stress response, potentially contributing to inflammation. Additionally, PPI analysis implicated altered expression of a set of cytokines serving as key nodes in the dysregulated pathways. These included predominantly increased TGF‐β (central to immunoregulatory and anti‐inflammatory responses), along with increased IL‐12 (key in Th1 responses), decreased IL‐4 (key in Th2 responses), reduced IFN‐γ and IFN‐β (critical for cell‐mediated and antiviral Th1 responses), and altered IL‐1 (involved in pro‐inflammatory responses). This pattern is indicative of immune dysregulation with chronic low‐grade inflammation.

This immune profile – combining MHC Class I deficits with a cytokine pattern indicative of chronic low‐grade inflammation – may be compatible with a latent chronic viral infection. This scenario may involve a combination of Th1 shift and cell‐mediated antiviral immune response (increased IL‐12 and decreased IL‐4), immune evasion (dampening IFN‐γ and IFN‐β production through viral interference with signaling pathways), immune exhaustion (progressive functional decline of antiviral immune cells and factors), and compensatory regulatory responses (increased TGF‐β to limit excessive inflammation), collectively hindering effective control of viral replication.^26^, ^27^, ^28^, ^29^, ^30^

Such an immune profile could also be compatible with autoimmune processes, as chronic viral infections can trigger or sustain autoimmunity through several mechanisms: molecular mimicry (structural similarity between viral and self‐antigens may lead to cross‐reactive immune responses), bystander activation (persistent inflammation may non‐specifically activate autoreactive lymphocytes), epitope spreading (tissue damage may unveil previously hidden self‐antigens, broadening the autoimmune response), and persistent immune activation (ongoing low‐level stimulation by latent viruses may contribute to chronic immune dysregulation).^31^

Consistent with these data and arguments, prior research has demonstrated a robust association between BD and chronic low‐grade inflammation (typically occurring in the absence of overt systemic illness or clinical inflammatory symptoms).^10^, ^11^ Notably, BD has been strongly linked to cytomegalovirus and *Toxoplasma gondii* (along with other herpesviruses, albeit less consistently, such as herpes simplex virus type 1, human herpesvirus 6, and Epstein–Barr virus), with multiple studies reporting increased seropositivity in affected individuals.^32^, ^33^, ^34^, ^35^, ^36^, ^37^ Finally, BD has been associated with signs of autoimmunity in a subgroup of patients, such as elevated levels of circulating auto‐antibodies (and in a small subset, even oligoclonal bands in the cerebrospinal fluid).^32^, ^38^ Moreover, our prior work demonstrated a reduction in circulating differentiated effector CD8+ T cells, alongside an increase in naïve CD4+ T cells in BD, closely resembling the T cell pattern observed in multiple sclerosis, a prototypical autoimmune disease.^39^ Interestingly, this pattern may reflect tissue migration or exhaustion of cytotoxic CD8+ T cells – mechanisms that further support a link with MHC Class I dysfunction^23^ and are observed in autoimmune conditions and, in some cases, in latent chronic viral infections, particularly when accompanied by immune dysregulation.^28^, ^39^

However, whether and how this systemic immune pattern plays a key role in the pathophysiology of BD remains largely unclear. Notably, our prior work has reported a correlation between reduced circulating differentiated effector CD8+ T cells and microstructure alterations in the brain's white matter in BD.^39^ Coherently, preliminary findings have also reported autoreactivity against myelin antigens and T cell infiltration into the brain parenchyma in individuals with BD.^40^, ^41^ These data suggest a potential link between immune dysregulation and immune‐mediated white matter damage, a prominent feature of BD,^5^, ^6^, ^7^ but further research is needed to clarify the role of these mechanisms in the disorder's pathophysiology.

### Limitations and future directions

Several important limitations and considerations should be noted when interpreting these findings and outlining directions for future research.

First, this is a proof‐of‐concept study with a small sample size. However, the proteomic analysis was specific to the platelet proteome – minimizing interference from abundant plasma or serum proteins and therefore enhancing the specificity of the analysis – and was systematic, data‐driven, and in‐depth. Moreover, the manic sample was clinically homogeneous and carefully matched 1:1 with HC for age and sex. Nonetheless, replication in larger cohorts is necessary.

Second, the analysis focused exclusively on the manic phase, limiting conclusions about the relationship between the observed immune correlates and psychopathology. In this context, TGF‐β levels were associated with mania, with platelet‐derived levels (increased in manic patients) possibly reflecting more stable links with the manic state, whereas plasma‐derived levels (correlated with mood elevation) possibly tracking fluctuations in manic symptom severity. Given the exploratory and hypothesis‐generating nature of this study, this pattern should be tested in future work using a dedicated design. Importantly, while mania may represent the core of BD, future studies should include depressive and euthymic phases – ideally with longitudinal designs – to assess the state‐ versus trait‐like nature of the observed alterations. Furthermore, the identified immune changes may not be specific to BD and could overlap with findings in other conditions, such as schizophrenia or major depressive disorder, underscoring potential shared pathophysiological processes across major psychiatric disorders and highlighting the need for future cross‐diagnostic investigations.

Third, the observational and correlational design of this study limits the ability to draw causal inferences regarding the role of the observed biological alterations in the underlying pathophysiology. The primary finding of this work is the detection, in mania, of deficits in MHC Class I–related pathways, along with potentially linked immune alterations. Because MHC alterations have been robustly linked to viral and autoimmune susceptibility in prior studies, including mechanistic work in animal models,^23^, ^24^, ^25^ we hypothesize that such deficits may contribute to viral, autoimmune, and inflammatory mechanisms in the pathophysiology of BD, forming the main focus of our discussion. However, beyond immune findings, we also observed alterations in pathways related to cell maturation and homeostasis – particularly those involving neural processes (e.g., SNARE signaling and synaptogenesis) – which may interact with MHC‐related changes and suggest potential neurodevelopmental and synaptic vulnerabilities. Additional alterations were detected in neurotransmitter signaling (e.g., serotonin pathways) as well as in multiple metabolic processes, further indicating that the observed changes extend beyond immune pathways to broader aspects of cellular and, possibly, neuronal function. Notably, MHC Class I deficits have also been mechanistically linked to increased long‐term potentiation and/or reduced long‐term depression, accompanied by decreased synaptic pruning, across several brain regions, particularly sensory cortices and the hippocampus; these alterations have been associated with hyperexcitable, less flexible neural networks and impaired activity‐dependent refinement and learning.^42^, ^43^, ^44^ Thus, although our findings derive from peripheral platelet proteomics, they may indirectly point to related central mechanisms. Specifically, neuroimmune alterations involving MHC pathways could influence synaptic plasticity and/or neuronal resilience to viral or immune‐related insults in BD. Therefore, our findings generate hypotheses regarding multiple pathophysiological mechanisms in BD, converging on immune and neuroimmune dysregulation centered on MHC deficits and related immune‐inflammatory alterations, along with possible synaptic dysfunction. Future targeted work – particularly in appropriately designed animal models and translational studies incorporating longitudinal assessment of these neuroimmune factors – may directly test these hypotheses, clarify their causal relationships, and determine whether such processes contribute to the pathophysiology of BD.

Additionally, beyond serving as peripheral markers of systemic and, possibly, brain‐related changes, platelets might play a more direct role in BD pathophysiology, particularly within the context of blood–brain crosstalk.^15^, ^17^ Although anucleate, platelets are increasingly recognized for their roles in immune surveillance, interactions with leukocytes, and the presentation or release of immunomodulatory molecules such as TGF‐β and CD40L.^17^ Future work should therefore examine whether specific platelet‐derived signals, including extracellular vesicles or surface markers, contribute functionally to neuroimmune communication or serve as reliable peripheral indicators of disease state or treatment response.

An emerging question concerns the possible upstream drivers of the potential MHC pathway deficits and related immune changes in BD. Genetic or epigenetic variations within the MHC region or associated regulatory pathways represent one plausible contributor, given the locus's immunogenetic complexity. Alternatively, environmental exposures inducing chronic inflammation could secondarily affect MHC signaling and downstream cytokine or synaptic processes. Future studies combining genomic, epigenomic, environmental, and longitudinal immune profiling will be required to confirm and clarify the relative contributions of these potential etiological mechanisms.

Finally, mechanistic elucidation of the potential role of MHC Class I deficits and related immune‐inflammatory alterations in BD may reveal targets, potentially at one or more points within the broader network of immune dysregulation, for the future development of more effective, disease‐centered therapeutic strategies.

### Conclusions

Taken together, this study highlights the potential of platelet proteomics as a valuable tool for investigating biological alterations in psychiatric disorders, including BD. Our findings reveal deficits in MHC Class I‐related pathways that align with chronic low‐grade inflammation and suggest potential roles for latent chronic viral infections and autoimmune mechanisms in BD. While further research is needed, these results reinforce the concept of immune dysregulation in mania and offer novel insights that may be integrated into existing biological frameworks. In particular, they may help refine the recently proposed unified model of the pathophysiology of BD, which links immune dysregulation to immune‐mediated white matter damage in limbic circuits, alterations in neurotransmitter signaling, reconfiguration of intrinsic brain activity, and the emergence of manic‐depressive psychopathology.^7^, ^8^, ^9^ Overall, these findings support the broader exploration of peripheral and central immune‐related processes as contributors to disease mechanisms, opening avenues for biomarker development and the delineation of mechanistic neuroimmune models in psychiatric disorders.

## Author contributions

P.M. and M.M. conceived the study, developed the theoretical framework, contributed to the interpretation of results, and wrote the manuscript. N.T.N.L. performed the proteomics data analysis, contributed to results interpretation, and drafted the methods and results sections, as well as the figures. W.‐Y.C. conducted subject recruitment, performed patient assessments, and collected clinical data. M.‐C.H. supervised subject recruitment and collection of clinical data. D.B. contributed to the study design and revised the manuscript. T.B. conceived and supervised the study, contributed to the interpretation of results, and revised the manuscript. P.M., M.M., W.‐Y.C., and T.B. secured funding for the study. All authors reviewed and approved the final version of the manuscript.

## Disclosure statement

The authors have no conflicts of interest to declare.

## Supporting information


**Table S1.** Relationship between TGF‐β1 levels and manic symptomatology.

## Data Availability

The datasets used and/or analyzed during the current study, as well as the full list of all quantified proteins and their related functions, are available from the corresponding author on reasonable request.
