# Supplementary material for: Immune dysregulation in mania: A proof‐of‐concept platelet proteomics study
Source: Psychiatry Clin Neurosci. 2026 Apr 4;80(7):575–84. doi: 10.1111/pcn.70055 (PMC13332576; doi:10.1111/pcn.70055)
Supplement: Supplementary file 1 — Table S1. Relationship between TGF‐β1 levels and manic symptomatology. [file PCN-80-575-s001.docx]

| **Supplementary Table 1. Relationship between TGF-β1 levels and manic symptomatology** | | |
| --- | --- | --- |
| **Manic symptomatology** | **Platelet TGF-β1** τ (p) | **Plasma TGF-β1** τ (p) |
| YMRS 1 (elevated mood) | 0.17 (0.52) | **0.63 (0.016)** |
| YMRS 2 (increased motor activity-energy) | -0.42 (0.11) | -0.07 (0.79) |
| YMRS 3 (sexual interest) | 0.09 (0.73) | 0.09 (0.73) |
| YMRS 4 (sleep) | 0.37 (0.15) | -0.04 (0.86) |
| YMRS 5 (irritability) | 0.04 (0.86) | 0.16 (0.54) |
| YMRS 6 (speech - rate and amount) | -0.17 (0.52) | 0.12 (0.64) |
| YMRS 7 (language-thought disorder) | 0.46 (0.08) | -0.30 (0.25) |
| YMRS 8 (content) | -0.04 (0.86) | -0.09 (0.73) |
| YMRS 9 (disruptive-aggressive behavior) | -0.32 (0.24) | 0.58 (0.03) |
| YMRS 10 (appearance) | 0.11 (0.68) | -0.03 (0.92) |
| YMRS 11 (insight) | -0.48 (0.07) | 0.05 (0.85) |
| YMRS total score | 0.04 (0.87) | 0.21 (0.39) |

Exploratory correlations between plasma and platelet TGF-β1 levels and YMRS scores in manic patients using two-tailed Kendall’s tau-b test. All items were inspected for outliers. No correction for multiple comparisons was applied due to the exploratory nature of the analysis. Plasma TGF-β1 is significantly correlated with YMRS Item 1 (elevated mood), highlighted in bold. No other correlations are significant, except for the correlation between plasma TGF-β1 and Item 9, which may be influenced by two outliers.

Abbreviation: YMRS, Young mania rating scale; TGF-β1, transforming growth factor beta 1.
